# The War in Gaza and Barriers to Inflammatory Bowel Disease Care

**DOI:** 10.1002/jgh3.70184

**Published:** 2025-05-14

**Authors:** Yotam Elimeleh, Ofer Ben‐Bassat, Ariel Benzvi, Eran Zittan

**Affiliations:** ^1^ The Abraham and Sonia Rochlin IBD Unit, Department of Gastroenterology and Liver Diseases Emek Medical Center Afula Israel; ^2^ The Rappaport Faculty of Medicine Technion‐Israel Institute of Technology Haifa Israel; ^3^ Barzilai Medical Center Ashkelon Israel

**Keywords:** financial difficulties, inflammatory bowel disease activity, patient‐reported outcomes, periphery‐residents, war‐time stress

## Abstract

**Objectives:**

Stress is associated with inflammatory bowel disease (IBD) development and exacerbation. We evaluated the impact of the war in Gaza on Israeli IBD patients and related barriers to IBD care.

**Methods:**

Adult IBD patients were blindly enrolled to complete a patient‐reported‐outcome electronic questionnaire assessing symptoms, hospitalizations, medications, psychosocial factors, economic issues, and adherence to therapy during wartime.

**Results:**

Overall, 526 participants completed the questionnaire, 67% with CD and 33% with UC. Fifty one percent had moderate–severe IBD patients. Compared to central residents, residents of peripheral regions described a higher need for financial support as their main missing aspect in IBD coping (26% vs. 17%), had increased financial difficulties attributed to wartime that led them to skip therapy (21% vs. 9%), and reported increased rates of requiring financial support to purchase biological medications (13% vs. 3%). Compared to mild patients, moderate–severe patients reported significantly more disease aggravations (47% vs. 23%), hospitalizations (16% vs. 2%), greater need for financial support as their main missing aspect in IBD coping (31% vs. 11%), increased financial difficulties attributed to wartime that led them to skip medical therapy (32% vs. 3%), increased rates of missing IBD medical therapy owing to wartime‐related stress (34% vs. 11%), and increased daily cannabis use (21% vs. 9%).

**Conclusions:**

Periphery‐residents with IBD experience more financial difficulties, hospitalizations, and disease exacerbation during wartime. Efforts should be taken to minimize disparities in medical care availability and accessibility, with special emphasis on moderate–severe patients who are more prone to disease aggravations.

**Abbreviations**
CCFI: Crohn's Colitis Foundation Israel CCFI
IBD‐PTS: IBD‐posttraumatic stress
PRO: patient reported outcome

## 1 Introduction

Mental stress, specifically relating to stressful major life events, as well as depressive and anxiety disorders, have been implicated in the development of inflammatory bowel diseases (IBD) [1, 2] through various complex brain‐gut interactions [3]. These stressors are also described as triggers for IBD exacerbations [4, 5, 6, 7], with current evidence suggesting that perceived stress situations precede IBD exacerbations [7, 8].

IBD patients have previously demonstrated markedly increased rates of posttraumatic symptoms, with around 20% suffering from posttraumatic stress disorder (PTSD) and up to one‐third suffering from IBD‐posttraumatic stress (IBD‐PTS), a less severe subset of PTSD. These conditions result in serious implications for patients' daily functioning and quality of life, causing occupational impairment, increasing the susceptibility to other psychiatric disorders including PTSD, and increasing the risk for CD exacerbations [9, 10, 11]. Currently identified predisposing factors to IBD‐PTS include the severity of IBD, negative hospitalization experiences, prior ileostomy surgery, and having Crohn's disease (CD) rather than ulcerative colitis (UC) as the primary diagnosis [9, 10, 12].

Specific literature and data regarding the activity of IBD during wartime stress is lacking.

On October 7th, 2023, during the Jewish holiday of Simchat Torah, thousands of Hamas terrorists invaded dozens of Israeli villages, Kibbutzim, and several cities near the border of Gaza in Central Israel. There they performed a massacre including raping, burning alive, and slaughtering entire families in their sleep including children. They also kidnapped over 200 civilians, including infants, and took them into the Gazan territory to hold them hostage. The events on October 7th represent the most severe hate crimes performed against the Jewish people since the Holocaust, leaving the entire Israeli society in horror and undermining the fundamental sense of safety in one's own home. Subsequently, Israel and its allies initiated a wide military operation against Hamas in the Gaza strip, with the primary aims of releasing all hostages and destroying the terrorist organization.

The main objective of this study was to evaluate the impact of wartime stress on Israeli IBD patients living within the more developed cities in central Israel contrasted with patients living in peripheral regions. Specifically, the war's impact on disease activity, patient‐reported outcomes (PROs), psychosocial and economic issues, as well as barriers to IBD therapy and adherence to therapy during the war against Hamas, conducted since October 2023.

## 2 Methods

This prospective cohort study took place between 21.2.2024 and 7.4.2024 and was approved by the institutional research ethics board (REB) of Emek medical center (Afula, Israel), approval number 007‐24‐EMC following a literature review and comprehensive analysis of current evidence, we formulated an electronic multiple‐choice questionnaire in Hebrew. The questionnaire consisted of several domains, each exploring a different area of interest. The first domain included sociodemographic questions addressing the patient's cultural background, area of residence, and economic issues concerning the current war period. The second domain assessed difficulties in adherence to IBD advanced therapy such as biological treatment and follow‐up during the war.

The third domain included questions evaluating current disease status through patient reported outcomes (PROs), regarding disease symptomatology and related psychosocial aspects. Patients were asked to rank their current symptoms on a Likert scale of 1 (no symptom at all) to 5 (symptom very severe). Symptoms included abdominal pain, diarrhea, fatigue, malnutrition, adherence to medical treatment, general quality of life, physical limitation in going to work, missed workdays, reduced ability to go outside home, IBD exacerbations (patients were asked to report subjectively, both generally about having disease exacerbation, and more specifically about increased stool frequency, abdominal pain and/or rectal bleeding), fistula activity, extraintestinal manifestations, psychological symptoms (mental stress, anxiety, and depression), and degree of damage to relationships.

In the fourth domain, patients were asked to answer the same questions as in the third domain but referring to their symptomatology and sensations 1 year before the war.

Inclusion criteria were age more than 18 years; initial IBD diagnosis at more than 18 years of age; follow up in a specialized IBD center for at least 12 months before October 2023.

Patients currently under biological, small molecule therapy, combination therapy, or who received more than 2 courses of corticosteroid therapy in the last 12 months were predefined as having moderate–severe IBD.

The primary outcome was changes in patient reported outcomes (PROs) and disease symptomatology, including subjective disease exacerbation owing to war‐time stress.

The main secondary outcomes were economic issues and difficulties owing to wartime that affect the ability to purchase IBD medications; differences in peripheral versus central residents in PROs, hospitalizations, and in difficulties in purchasing medications.

The questionnaire was electronically sent via E‐mail to a total of 895 adult IBD patients (≥ 18 years old) diagnosed with either CD or UC as their primary diagnosis, which were blindly enrolled from the Crohn's Colitis Foundation Israel (CCFI) database and from two specialized IBD units in Israel. Peripheral (northern and southern) residence and central residence were defined by the patient's addresses as the least and most densely populated Israeli regions, respectively, derived from the Israeli central bureau of statistics [13].

### 2.1 Statistical Analysis

Continuous variables were presented as either the mean ± SD or the median with the interquartile range (IQR) depending on whether the data approximated a normal distribution. Differences between groups were assessed using the Student's *t*‐test for normally distributed variables and the Mann–Whitney *U* test for non‐normally distributed variables. For all analyses, the statistical significance level was defined as *p* < 0.05. All statistical analyses were performed using SPSS (version 21.0, SPSS Inc., Chicago, IL, USA).

## 3 Results

A total of 526 participants completed the questionnaire, 354 (67%) with CD and 172 (33%) with UC as their primary diagnosis. Of the patients that completed the questionnaire, 51% had moderate–severe disease (*n* = 266), and 49% had mild disease (*n* = 260). In total, 37% (*n* = 193) of participants reported having ≥ 1 relative directly affected by the war (injured, killed, or kidnapped). Additionally, 36% of all participants (*n* = 187) reported disease aggravations compared to the previous year, primarily expressed as increased fatigue, increased severity of mental symptoms, and increased frequency of bowel movements (data not shown). Furthermore, 37% (*n* = 194) of all participants reported increased severity in mental symptoms (anxiety, depression, and mental stress) during the wartime compared to the previous year. Fifty‐nine percent (*n* = 310) of respondents lived in peripheral regions of Israel, and 41% (216) lived in central regions of Israel.

The baseline demographic and general characteristics of respondents compared between periphery and non‐periphery residents, are presented in Table 1. Both groups were comparable in mean disease duration, in the distribution of age, gender, and in their primary IBD diagnosis (CD vs. UC), as well as in having at least one relative directly affected by the war (injured, killed, or kidnapped).

**TABLE 1:** Demographic and general characteristics of participants, periphery residents versus non‐periphery (central) residents.

	Periphery residents (*n* = 310, 59%)	Non‐periphery residents (*n* = 216, 41%)	*p*
Mean age (years)	44.7 (±15.3)	47.1 (±16.8)	*p* = 0.03
Sex	Male—156 (50.2%) Female—154 (49.8%)	Male—88 (41%) Female—127 (59%)	*p* = 0.03
Primary diagnosis (CD vs. UC)	CD—217 (70%) UC—93 (30%)	CD—137 (63%) UC—79 (37%)	*p* = 0.20 *p* = 0.07
Moderate–severe disease patients^a^	118 (74%) *N* = 159	64 (60%) *N* = 107	*p* = 0.01
Mean disease duration (years)	14.2 (±11.0)	17 (±12.2)	*p* = 0.005
A lower‐than average monthly income	94 (30%)	46 (21%)	*p* = 0.03
Having ≥ 1 relative directly affected by the war (injured/killed/kidnapped)	116 (37%)	77 (36%)	*p* = 0.66

Abbreviations: CD, Crohn's disease; UC, ulcerative colitis.

^a^ Patients currently under advanced therapy such as biologicals, small molecule therapy, combination therapy, or who were treated with more than 2 cycle of steroid in the last 12 months were pre‐defined as having moderate–severe IBD.

Among periphery‐residents there were significantly more steroid users and patients under biological therapy compared with non‐periphery (central) residents (74% vs. 60%, respectively, *p* = 0.01). Additionally, significantly more periphery residents had a lower‐than‐average income with respect to central residents (30% vs. 21%, respectively, *p* = 0.03).

Table 2 presents a subgroup analysis of war‐related psychosocial issues and barriers to IBD therapy, and adherence among periphery residents compared with central residents. Six percent of periphery residents were evacuated from their homes during the war due to security issues, and none of the central residents were evacuated from home during the war (*p* = 0.001). Compared to central residents, periphery residents described the need for financial support as their main missing aspect in daily coping with IBD (26% in periphery vs. 17% in non‐periphery residents, *p* = 0.04). Periphery residents also had significantly increased financial difficulties attributed to wartime that led them to skip medical therapy (21% vs. 9% respectively, *p* = 0.04), and reported increased rates of requiring financial support to purchase advanced therapy such as biological medications (13% vs. 3%, respectively, *p* = 0.004). No significant differences were found between periphery and central residents in disease aggravation compared to the prior year, in hospitalization rates since war began, or in missing therapy owing to wartime stress.

**TABLE 2:** War‐related psychosocial issues and barriers to IBD therapy and adherence among periphery residents compared with non‐periphery (central) residents.

	Periphery residents (*n* = 310, 59%)	Non‐periphery residents (*n* = 216, 41%)	*p*
Describing disease aggravation compared to the year before	109 (35%)	78 (36%)	*p* = 0.82
Need for financial support as the main missing aspect in daily coping with IBD^a^	52 (26%) *N* = 202	24 (17%) *N* = 145	*p* = 0.04
Having financial difficulties attributed to wartime that have rendered me to skip medical therapy^b^	14 (21%) *N* = 101	4 (6%) *N* = 67	*p* = 0.04
Requiring financial support to purchase advanced therapy such as biological medications^b^	25 (13%) *N* = 194	4 (3%) *N* = 121	*p* = 0.004
Hospitalized since war began	31 (10%)	18 (8%)	*p* = 0.52
Occasionally missing IBD medical therapy owing to wartime‐related stress, concerns and lack of concentration^b^	57 (56%) *N* = 101	45 (67%) *N* = 67	*p* = 0.07
Being evacuated from one's home during the war due to security issues	18 (6%)	0	*p* = 0.001

Abbreviation: IBD, inflammatory bowel disease.

^a^ Among those who mentioned there are missing tools to deal with the disease.

^b^ Among those who indicated that they miss treatments.

Table 3 presents a subgroup analysis of war‐related psychosocial issues and barriers to IBD therapy among moderate–severe IBD patients, compared with mild IBD patients. Moderate–severe patients had significantly increased rates of disease aggravation compared to the previous year (47% vs. 23%, *p* < 0.0001), and increased hospitalization rates since war began (16% vs. 2%, *p* < 0.0001) compared to mild patients. Regarding financial difficulties, moderate–severe patients described a significantly greater need for financial support as their main missing aspect in daily coping with IBD when compared to mild patients (31% vs. 11%, respectively, *p* < 0.0001), and had significantly increased financial difficulties attributed to wartime that led them to skip medical therapy (32% vs. 3%, respectively, *p* < 0.0001). Furthermore, moderate–severe patients had increased rates of missing IBD medical therapy owing to wartime‐related stress, concerns, and a lack of concentration (34% vs. 11%, *p* = 0.0002), and had substantially increased rates of daily cannabis use (21% vs. 9%, *p* = 0.0002). No significant difference was demonstrated between the two groups in access to medical care due to evacuation from home.

**TABLE 3:** War‐related psychosocial issues and barriers to IBD therapy and adherence among moderate–severe IBD compared with mild IBD.

	Moderate–severe IBD^c^ (*n* = 266, 51%)	Mild IBD (*n* = 260 49%)	*p*
Having ≥ 1 relatives directly affected by the war (injured/killed/kidnapped)	96 (36%)	97 (37%)	*p* = 0.75
Describing disease aggravation compared to the year before	126 (47%)	61 (23%)	*p* < 0.0001
Need for financial support as the main missing aspect in daily coping with IBD^a^	59 (31%) *N* = 192	17 (11%) *N* = 155	*p* = 0.0001
Having financial difficulties attributed to wartime that have rendered me to skip medical therapy^b^	24 (32%) *N* = 74	3 (3.2%) *N* = 94	*p* < 0.0001
Required financial support to purchase advance therapy such as biological medications	21 (20%) *N* = 106	8 (4%) *N* = 209	*p* = 0.47
Hospitalized since war began	43 (16%)	6 (2%)	*p* < 0.0001
Occasionally missing IBD medical therapy owing to wartime‐related stress, concerns and lack of concentration^b^	25 (34%) *N* = 74	10 (11%) *N* = 94	*p* = 0.0002
Reduced access to medical care because I was evacuated from my home since war began^b^	1 (1.3%) *N* = 74	2 (2%) *N* = 94	*p* = 0.71
Daily cannabis use	55 (21%)	24 (9%)	*p* = 0.0002

Abbreviation: IBD, inflammatory bowel disease.

^a^ Among those who mentioned there are missing tools to deal with the disease.

^b^ Among those who indicated that they miss treatments.

^c^ Patients currently under advanced therapy such as biologicals, small molecule therapy, combination therapy, or who were treated with more than 2 cycle of steroid in the last 12 months were pre‐defined as having moderate–severe IBD.

## 4 Discussion

In this study we have demonstrated significantly more financial difficulties during wartime among residents living in the periphery of Israel compared to residents of central Israel, and substantially increased rates of disease exacerbations and hospitalization, as well as financial difficulties, as reported by the patients (PROs), among moderate–severe IBD patients compared to mild IBD patients, during the current war in the Gaza strip.

Wars and other types of armed conflicts are obvious environmental stressors for civilians on both sides of the conflict and can carry disastrous implications on a multitude of public services in various ways, particularly on local healthcare systems. This includes disruption of daily life, increasing stress, and predisposing individuals to major psychiatric disorders [14], by destruction of public buildings and infrastructure, including medical care centers and hospitals, and by diversion and depletion of resources to the combative effort [15, 16]. Particularly in the less developed peripheral and rural regions, these effects may lead to a collapse of healthcare systems [15, 16]. Altogether, these war effects markedly limit access to medications and other health services, with detrimental consequences for patients suffering chronic diseases, including IBD. Consequentially, this reduces patient adherence to medical care and follow up, interrupting treatment, and dramatically increasing the exacerbations and burdens of chronic disease, leading to an increase in overall morbidity and mortality [15, 17].

The results of this study are in accordance with previous data regarding the well described reduced access to medical care and healthcare facilities in rural and peripheral regions compared to central regions, with disparities in medical care access resulting from financial, cultural, and geographic limitations in these areas, potentially leading to poorer health outcomes and also reduce overall quality of life [18, 19]. Specifically regarding Israel, a recent large scale nationwide cohort study covering almost the entire Israeli population has shown that residence in peripheral regions and lower socioeconomic status were both significantly associated with worse IBD disease outcomes, namely steroid dependency and CD‐related surgery [20].

Despite Israel is a small country with a relatively close proximity between its central and peripheral regions, disparities in healthcare accessibility, quality and health outcomes between these regions are well known, have been previously addressed by the Israeli government, and are partially explained by socioeconomic regional differences [21, 22].

In Israel, healthcare services are public, and a health insurance law exists, by which all residents adult residents are obliged to pay to the national insurance institute for health insurance coverage, with the fee calculated by one's income and occupational status, and all insured persons must be registered as members of a health maintenance organization (HMO) that must provide them with a standard “health basket” specified by law and updated annually [21, 22]. As for IBD biological therapy, most of its cost is subsidized by the Israeli government ministry of health, but a significant amount of the cost is brought by patient participation.

The higher rates of disease exacerbation, hospitalizations and financial difficulties demonstrated in our study among patients with moderate–severe disease, and the actual absence of significant war‐related effect in patients with mild disease might be explained by the fact that patients with more severe disease generally require more complex professional medical approach, more complex and costly pharmacological therapy, and are more dependent on medical care for optimization of disease control, and therefore are more vulnerable to the effect of lack of medical services, as well as to the financial effects of wartime.

A potential explanation for the financial difficulties demonstrated in this study for moderate–severe patients is their advanced therapy, such as biological therapy, and small molecule therapy, which is dramatically more costly than non‐advanced therapy used to treat milder cases of IBD. The affordability of IBD care with only partial reimbursements, thereby limits access to medical care especially for moderate–severe patients [23]. Subsequently, limited affordability of advanced therapy may also reduce patient adherence and cause treatment interruption. This increases the potential for developing loss of response and infusion reactions to biological drugs [24, 25], and can also increase the frequency of IBD exacerbations. Altogether, limiting access to care because of affordability increases morbidity and markedly impairing quality of life [26].

Furthermore, during the current war around 150 000 people were evacuated and displaced from their homes in peripheral Israeli regions adjacent to the western border with the Gaza strip and to the northern border with Lebanon, and many lost their jobs [27]. This situation rendered a combination of economic difficulties and reduced healthcare service availability which we assume that greatly impacted IBD management, and can at least partially explain our results among periphery residents and moderate–severe IBD patients.

Our study has several limitations, including a relatively small sample size and the potential over‐representation of periphery‐residents. There was a higher percentage of moderate–severe patients within the periphery‐residents group, compared to the non‐periphery (central) residents group, which could potentially influence the results. Furthermore, PRO questionnaires often do not capture nor correlate with objective measurements of disease activity as well as IBD management practices, and may ineffectively represent the complexity of factors influencing disease activity and adherence to therapy, considering real‐life healthcare system constraints, scenarios in which guideline recommendations are not feasible, and other physician decision‐making processes. In addition, in our study the number of CD responders is twice that of UC responders, not reflecting the actual almost 1:1 ratio between CD and UC among Israeli IBD patients reported in the literature [28]. This latter limitation, together with the relatively small sample size in our study, has limited us in reaching significant conclusions by subgroup analysis comparing CD and UC, and therefore our findings were on IBD patients in general, and not specific for either of the specific IBD subtypes.

## 5 Conclusions

IBD patients who reside in peripheral regions, and moderate–severe IBD patients experience more financial difficulties, with increased hospitalizations and patient‐reported disease exacerbation in moderate–severe IBD patients during wartime stress. All efforts should be taken, especially during wartime, to minimize the differences in the availability and accessibility to medical care between IBD peripheral residents and central residents. Especially increasing the focus on moderate–severe patients who are more prone for disease aggravations. This would improve therapy adherence and prevent disease exacerbations by embracing a multidisciplinary and holistic approach in specialized IBD centers.

## Ethics Statement

This study was approved by the institutional research ethics board (REB) of Emek medical center, approval number 007‐24‐EMC and have therefore been performed in accordance with the ethical standards laid down in the 1964 Declaration of Helsinki and its later amendments.

## Conflicts of Interest

Eran Zittan has received research support and consulting fees from Janssen, AbbVie, Takeda, Neopharm, Celgene, and Pfizer. The other authors declare no conflicts of interest.
